# Essential minerals and risk of pancreatic diseases: a large-scale prospective cohort study

**DOI:** 10.3389/fnut.2026.1773339

**Published:** 2026-04-29

**Authors:** Pengwei Cao, Zhihua Shui, Xinyu Zhang, Endi Zhou, Tan Liang, Feng Cao, Chuang Yang, Daoxiang Zhang, Lei Liu, Jiangming Chen, Fubao Liu

**Affiliations:** 1 Hepatopancreatobiliary Surgery, Department of General Surgery, The First Affiliated Hospital of Anhui Medical University, Hefei, China; 2 School of Life Sciences, Anhui Medical University, Hefei, China; 3 Medical Faculty, University of RWTH Aachen, Aachen, Germany; 4 Medical Faculty, University of Leipzig, Leipzig, Germany

**Keywords:** essential minerals, pancreatic cancer, pancreatitis, pancreatitis risk factors, prospective study

## Abstract

**Objective:**

Pancreatic disorders are characterised by high mortality rates and limited therapeutic options and pose a global health challenge. As a dual-function organ with endocrine and exocrine roles, the pancreas exhibits a heightened sensitivity to the disruption of mineral homeostasis. However, the pathophysiological associations between mineral imbalances and pancreatic diseases remain controversial, and large-scale population studies validating these relationships are lacking.

**Methods:**

This prospective cohort study used data from the UK Biobank and enrolled 191,875 participants with a median follow-up of 13 years. A phenome-wide association study (PheWAS) was used to systematically evaluate the associations between mineral levels and multisystem disorders. Multivariate Cox proportional hazards regression models quantified risk associations, whereas restricted cubic spline analyses elucidated dose–response relationships.

**Results:**

PheWAS identified multiple significant associations within the digestive system, including cholelithiasis, gastritis/duodenitis, and pancreatic diseases. Elevated serum iodine and selenium levels demonstrated significant carcinogenic effects on pancreatic cancer. In contrast, copper, magnesium, and manganese exhibited protective effects against acute pancreatitis, with manganese displaying a U-shaped dose–response relationship. Subgroup analyses revealed increased iodine and selenium carcinogenicity in females, older individuals, and smokers, whereas metal-related protection was more pronounced in males and normal-weight individuals.

**Conclusion:**

Mineral homeostasis exerts systemic effects on digestive pathophysiology. Elevated iodine and selenium levels are modifiable risk factors for pancreatic carcinogenesis, particularly in females and older populations. The inverse association of copper, magnesium, and manganese with acute pancreatitis suggests that they are potential therapeutic targets. The attenuated association in chronic pancreatitis implies an etiological predominance of non-mineral mechanisms.

## 1 Introduction

Pancreatic pathologies, including pancreatic carcinoma, acute/chronic pancreatitis, and related disorders, are critical global public health concerns because of their elevated morbidity, escalating mortality rates, and constrained therapeutic alternatives (1–3). Functioning as a vital bihormonal organ that orchestrates endocrine and exocrine processes, the pancreas is particularly vulnerable to metabolic dysregulation (4, 5). Recent investigations have focused on the interplay between essential dietary mineral homeostasis and susceptibility to pancreatic disease; however, substantial controversies persist regarding the precise molecular mechanisms and clinical implications.

The evolving paradigm in pancreatology emphasises that environmental and nutritional determinants are modifiable risk factors in disease pathogenesis (6, 7). Essential minerals exhibit complex dual physiological roles. For example, despite its canonical antioxidant properties, elevated selenium concentrations may paradoxically promote carcinogenesis in specific tissue microenvironments (8); beyond its thyrotropic functions, iodine appears to exert pancreas-specific regulatory effects (9, 10). This biological complexity necessitates a systematic investigation of mineral–pancreas interactions. However, previous studies predominantly focused on single-element analyses and had restricted sample sizes and marked heterogeneity across findings. For instance, although oxidative stress is mechanistically implicated in both acute and chronic pancreatitis (11), conflicting evidence exists regarding copper-mediated potentiation (12) versus magnesium-dependent suppression (13) of this pathway, with a paucity of large-scale validation in clinical populations. These knowledge gaps highlight the importance of comprehensive prospective studies elucidating mineral–disease associations.

Therefore, the aim of this prospective cohort study quantitatively was to evaluate the risk associations between essential minerals, including calcium, magnesium, iron, zinc, copper, potassium, phosphorus, selenium, and pancreatic disease spectrum using the UK Biobank’s large-scale population-based data.

## 2 Methods

### 2.1 Study design and population

This study was based on data from the UK Biobank, which is a large-scale population-based prospective cohort study. Between 2006 and 2010, over 500,000 participants aged 37–73 years were recruited from 22 assessment centres across England, Scotland, and Wales. At baseline, comprehensive data were collected using touchscreen questionnaires, face-to-face interviews, physical examinations, and biological sample collection. The participants’ health outcomes were continuously monitored using electronic health records, including hospital inpatient data, cancer registries, and death records. All participants provided informed electronic consent, and the study was approved by the North West Multi-Centre Research Ethics Committee (14).

### 2.2 Assessment of essential mineral exposure

The participants were selected based on the availability of dietary data. Between 2009 and 2012, the UK Biobank conducted five rounds of web-based 24-h dietary recall assessments (Oxford WebQ), which were sent via email to participants who provided valid email addresses. Of the >250,000 invited individuals, 210,025 completed the dietary questionnaire at least once. The number of responses per round ranged from 69,551 to 102,958. Each participant completed an average of 2.17 dietary assessments. The current study only included participants with available data from at least one dietary assessment and used the integrated results across all five rounds to estimate long-term dietary intake. Dietary intake of essential minerals, including calcium, magnesium, iron, zinc, copper, potassium, phosphorus, and selenium, was determined by trained nutritionists based on self-reported food consumption using the UK Nutrient Databank. The daily intake values for each mineral were calculated as the mean intake across all available assessments per participant, providing a stable estimate of habitual intake.

### 2.3 Outcome assessment

The primary outcome of this study was the incidence of pancreatic diseases, including cancer, acute pancreatitis, chronic pancreatitis, and other related conditions. These outcomes were identified based on linkages to electronic health records and were defined using the International Classification of Diseases, 10th Revision (ICD-10) codes: C25 for pancreatic cancer, K85 for acute pancreatitis, and K86 for chronic pancreatitis and other pancreatic disorders. Participants were followed up from baseline until the first occurrence of pancreatic disease, death, or 31 October 2022, whichever occurred first.

### 2.4 Covariate assessment

Baseline sociodemographic and lifestyle information was collected via touchscreen questionnaires, including age, sex, ethnicity (categorised as white or non-white), and socioeconomic status, which were assessed using the Townsend Deprivation Index (TDI) and annual household income before tax. Smoking status and alcohol consumption were classified as never, previous, or current. Physical activity was quantified using the Metabolic Equivalent of Task (MET) minutes per week, including walking, moderate, and vigorous activities (15). Height and weight were measured to calculate body mass index (BMI). All blood samples were collected at baseline, and the fasting time was recorded at the time of sampling. Biochemical measurements were performed within 24 h and included triglyceride, fasting plasma glucose, glycated haemoglobin, total cholesterol, and high- and low-density lipoprotein cholesterol levels. The prevalence of diabetes mellitus and dyslipidaemia was also assessed.

### 2.5 Phenome-wide association study analysis

A phenome-wide association study (PheWAS) was conducted to systematically evaluate the associations between the dietary intake of essential minerals and various health outcomes. Clinical diagnoses were coded using ICD-10 codes based on electronic health records from hospital inpatient data and death registries. All available ICD-10 codes were extracted for each participant, duplicates were removed, diagnoses reported postmortem were included, and the primary causes of death were determined. The PheWAS package was used to map the ICD-10 codes to 1,813 unique PheCodes, representing clinically meaningful disease groupings. Separate generalised linear models were fitted for minerals using each PheCode as the binary outcome (case/control) and standardised mineral intake (*Z*-score) as exposure. Each model yielded a *β* coefficient and associated *p*-value. Only PheCodes with at least 20 recorded cases were included to ensure statistical reliability. All models were adjusted for key covariates, including age, sex, BMI, and ethnicity. These covariates were included to account for the potential confounding factors.

### 2.6 Statistical analyses

We conducted normality tests on all continuous variables (e.g., age, BMI, MET, and mineral intake) using statistical methods, including the Kolmogorov–Smirnov test and the Shapiro–Wilk test. Due to the large sample size (*N* = 191,875), even minor deviations from normality resulted in statistically significant test results, which is expected in large-scale epidemiological studies. Continuous variables are described as means with standard deviations, and categorical variables are expressed as counts and percentages. All exposure variables (daily intake of essential minerals) were standardised as *Z*-scores before analysis.

Cox proportional hazards regression models were used to estimate the hazard ratios (HRs) and 95% confidence intervals (CIs) for the association between each standard deviation increase in essential mineral intake and the risk of pancreatic diseases. The proportional hazard assumption was tested using Schoenfeld residuals. Two models were constructed: model 1 was unadjusted, whereas model 2 was adjusted for age, sex, ethnicity, BMI, TDI, annual household income, MET, smoking status, alcohol consumption, diabetes, and dyslipidaemia. Restricted cubic spline (RCS) regression was performed using knots at the 10th, 50th, and 90th percentiles of mineral intake to assess potential nonlinear dose–response relationships.

## 3 Results

### 3.1 Baseline characteristics of study population

**Figure 1:**
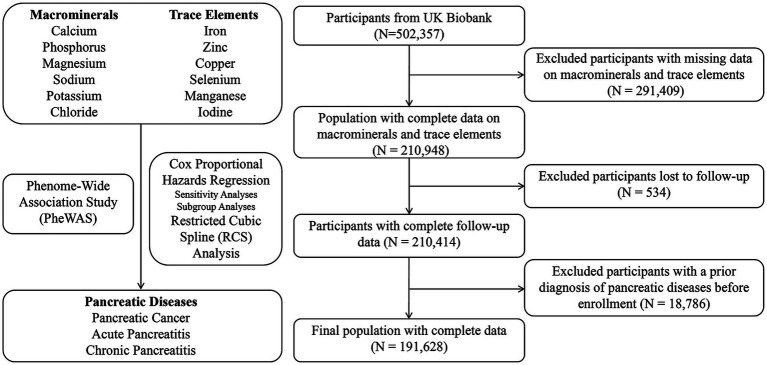
Flowchart illustrating the overall design and steps of the study.

The overall study design and participant selection processes are outlined in Figure 1. A total of 191,875 participants were included in the analysis, comprising 189,709 individuals in the control group, 249 with pancreatic cancer, 944 with acute pancreatitis, and 973 with chronic pancreatitis or other pancreatic conditions. Significant differences in baseline characteristics were observed among the four groups. Participants with pancreatic cancer were the oldest on average (61.07 ± 5.94 years), included a higher proportion of males (52.2%), and exhibited a significantly higher BMI than the control group (27.81 vs. 26.95, *p* < 0.001). In terms of socioeconomic status, individuals with pancreatic disease had higher TDI scores and a greater proportion of low-income households. Furthermore, the prevalence of smoking, alcohol consumption, and diabetes was significantly higher among participants with pancreatic disease than in the control group (all *p* < 0.001) (Table 1).

**Table 1:** Baseline characteristics of participants.

Characteristics	Normal (*N* = 189,709)	Pancreatic cancer (*N* = 249)	Acute pancreatitis (*N* = 944)	Chronic pancreatitis and others (*N* = 973)	*p* value
Age	55.77 (7.96)	61.07 (5.94)	57.90 (7.79)	60.18 (6.83)	<0.001
Sex					0.006
Female	102,696 (54.1)	119 (47.8)	482 (51.1)	473 (48.6)	
Male	87,013 (45.9)	130 (52.2)	462 (48.9)	500 (51.4)	
Ethnicity					0.45
White	180,532 (95.2)	238 (95.6)	904 (95.8)	936 (96.2)	
Mixed	1,160 (0.6)	3 (1.2)	4 (0.4)	2 (0.2)	
Asian or Asian British	3,395 (1.8)	3 (1.2)	21 (2.2)	16 (1.6)	
Black or Black British	2,478 (1.3)	1 (0.4)	5 (0.5)	7 (0.7)	
Others	2,144 (1.1)	4 (1.6)	10 (1.1)	12 (1.2)	
Body mass index	26.95 (4.64)	27.81 (5.05)	29.18 (5.64)	27.79 (4.92)	<0.001
Townsend deprivation index	−1.56 (2.88)	−1.61 (2.96)	−1.16 (2.96)	−1.34 (2.93)	<0.001
Annual household income before tax, £					<0.001
<18,000	19,491 (10.3)	32 (12.9)	101 (10.7)	128 (13.2)	
18,000-30,999	25,858 (13.6)	50 (20.1)	192 (20.3)	208 (21.4)	
31,000–51,999	40,589 (21.4)	57 (22.9)	225 (23.8)	270 (27.7)	
52,000-100,000	48,745 (25.7)	61 (24.5)	245 (26.0)	202 (20.8)	
>100,000	42,386 (22.3)	34 (13.7)	155 (16.4)	122 (12.5)	
Unknown	12,640 (6.7)	15 (6.0)	26 (2.8)	43 (4.4)	
MET	878.22 (1,037.00)	969.08 (1,144.67)	831.35 (983.83)	941.78 (1,062.69)	0.038
Smoking status					<0.001
Never	107,861 (56.9)	116 (46.6)	444 (47.0)	444 (45.6)	
Previous	66,421 (35.0)	102 (41.0)	402 (42.6)	412 (42.3)	
Current	14,923 (7.9)	31 (12.4)	91 (9.6)	113 (11.6)	
Unknown	504 (0.3)	0 (0.0)	7 (0.7)	4 (0.4)	
Alcohol status					0.003
Never	6,177 (3.3)	10 (4.0)	38 (4.0)	46 (4.7)	
Previous	5,674 (3.0)	15 (6.0)	34 (3.6)	37 (3.8)	
Current	177,664 (93.7)	224 (90.0)	869 (92.1)	888 (91.3)	
Unknown	194 (0.1)	0 (0.0)	3 (0.3)	2 (0.2)	
Diabetes					<0.001
No	182,249 (96.1)	225 (90.4)	886 (93.9)	880 (90.4)	
Yes	7,460 (3.9)	24 (9.6)	58 (6.1)	93 (9.6)	
Hyperlipemia					0.47
No	114,853 (60.5)	145 (58.2)	551 (58.4)	588 (60.4)	
Yes	74,856 (39.5)	104 (41.8)	393 (41.6)	385 (39.6)	

Values are presented as mean (SD) for continuous variables and *n* (%) for categorical variables. MET: metabolic equivalent of task.

### 3.2 PheWAS of essential minerals with multisystem diseases

**Figure 2:**
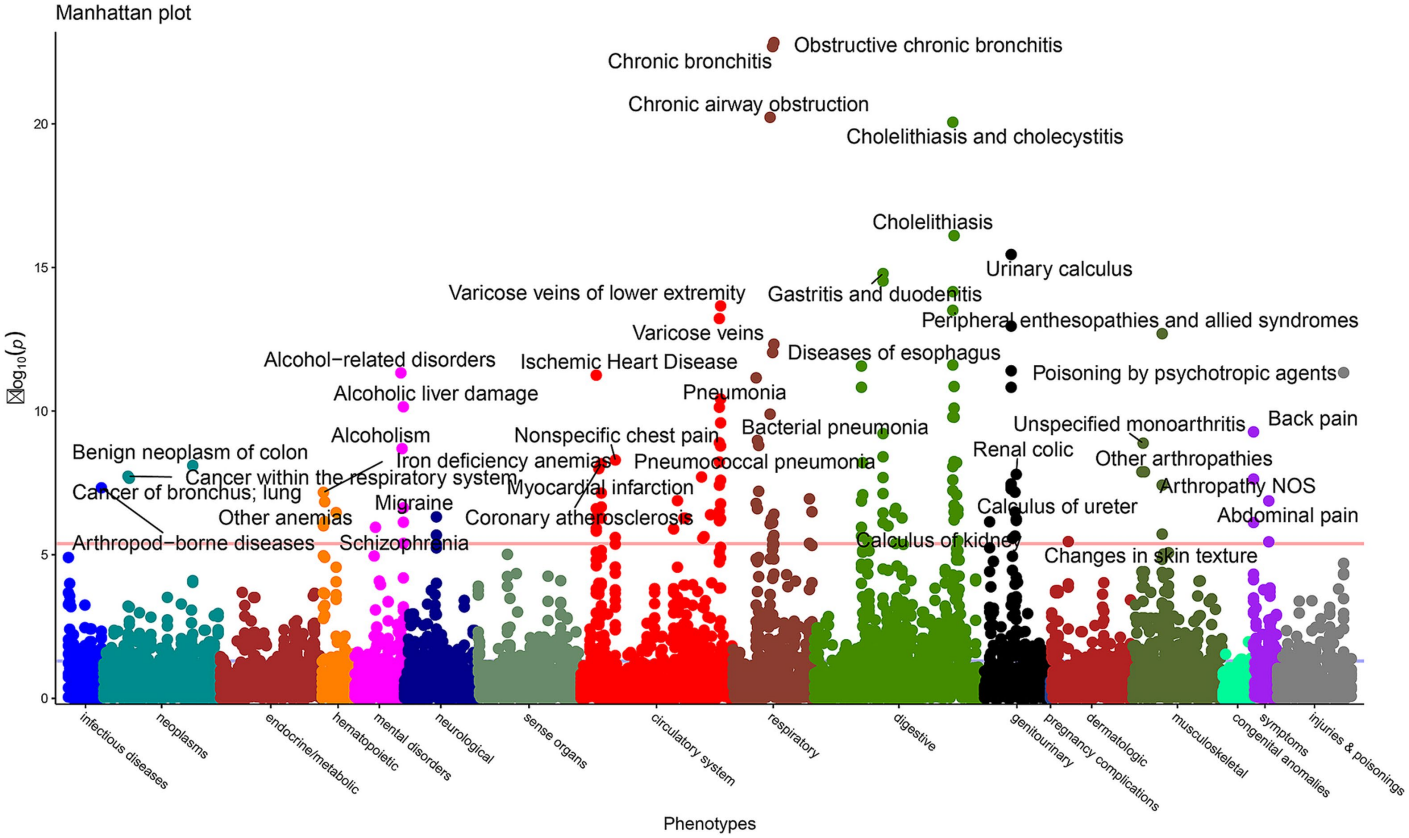
Phenome-wide association study (PheWAS) exploring the associations between essential minerals and a broad spectrum of diseases.

A comprehensive PheWAS was conducted using data from the 191,875 participants to evaluate the relationships between essential minerals and a broad spectrum of clinical phenotypes. This analysis spanned multiple organ systems, including infectious diseases, neoplasms, metabolic disorders, cardiovascular diseases, respiratory diseases, gastrointestinal disorders, genitourinary diseases, musculoskeletal conditions, and neuropsychiatric disorders. Essential minerals were significantly associated with numerous systemic diseases. In the cardiovascular domain, significant associations were observed between ischaemic heart disease, myocardial infarction, and coronary atherosclerosis (all *p* < 0.001). Within the respiratory system, significant associations were identified with bronchial/lung cancer, chronic obstructive pulmonary disease, and bronchitis (all *p* < 0.001). Additionally, multiple significant signals were detected in digestive disorders, including cholelithiasis, gastritis, duodenitis, and pancreatic diseases, as well as in metabolic diseases, such as iron deficiency anaemia and other types of anaemia (all *p* < 0.001) (Figure 2).

### 3.3 Associations between essential minerals and risk of pancreatic diseases

In the unadjusted model (Model 1), higher iodine (HR = 1.17; 95% CI: 1.07–1.28; *p* < 0.001) and selenium levels (HR = 1.12; 95% CI: 1.01–1.24; *p* = 0.028) were significantly associated with an increased risk of pancreatic cancer (Table S1). In contrast, copper (HR = 0.91; 95% CI: 0.85–0.98; *p* = 0.008), iron (HR = 0.93; 95% CI: 0.87–1.00; *p* = 0.040), magnesium (HR = 0.91; 95% CI: 0.85–0.97; *p* = 0.004), and manganese (HR = 0.88; 95% CI: 0.82–0.94; *p* < 0.001) were inversely associated with the risk of acute pancreatitis. Elevated potassium levels were positively associated with the risk of chronic pancreatitis and other pancreatic conditions (HR = 1.08; 95% CI: 1.02–1.15; *p* = 0.007).

**Figure 3:**
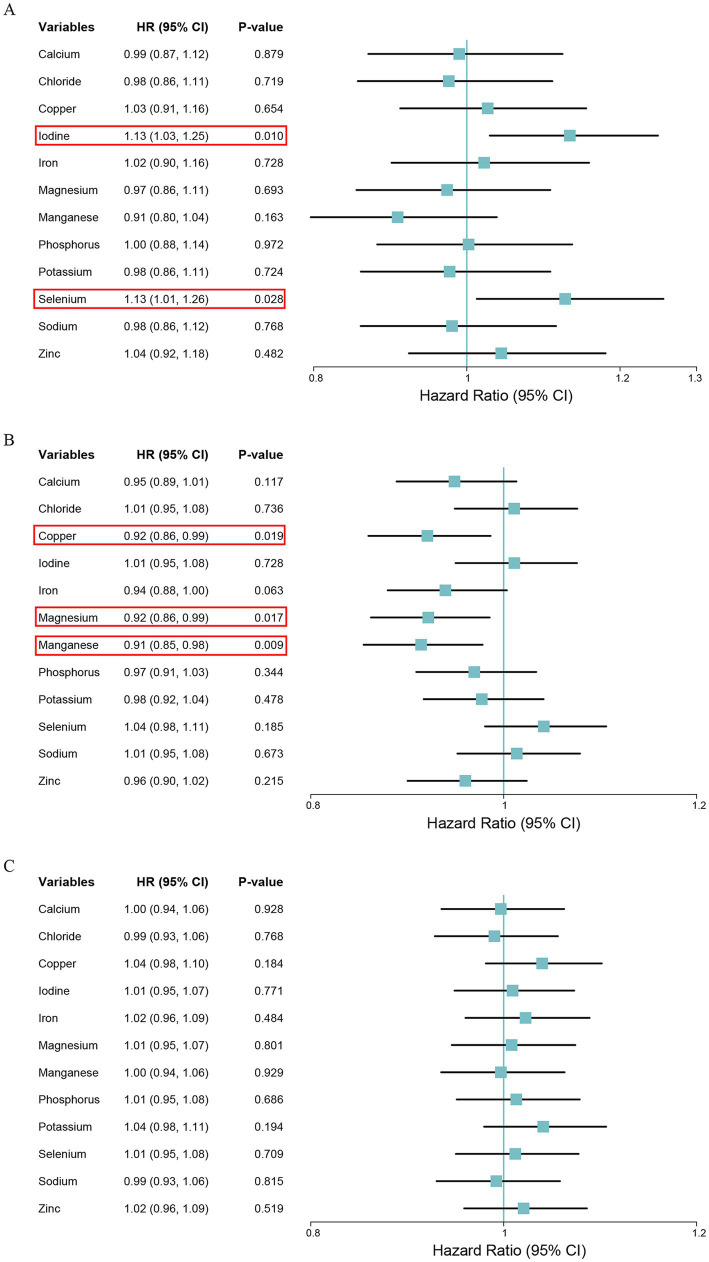
Associations between essential minerals and the risk of pancreatic diseases based on multivariable-adjusted models. **(A)** Pancreatic cancer, **(B)** Acute pancreatitis, **(C)** Chronic pancreatitis and other pancreatic conditions. HR: hazard ratio; CI: confidence interval. Model 2 was adjusted for age, sex, ethnicity, body mass index (BMI), Townsend Deprivation Index (TDI), annual household income before tax, Metabolic Equivalent of Task (MET), smoking status, alcohol status, diabetes, and hyperlipidemia.

After adjusting for potential confounders in the multivariate model (model 2), several associations remained significant. Specifically, iodine (HR = 1.13; 95% CI: 1.03–1.25; *p* = 0.010) and selenium (HR = 1.13; 95% CI: 1.01–1.26; *p* = 0.028) continued to show positive associations with pancreatic cancer risk (Figure 3A). In addition, copper (HR = 0.92; 95% CI: 0.86–0.99; *p* = 0.019), magnesium (HR = 0.92; 95% CI: 0.86–0.99; *p* = 0.017), and manganese (HR = 0.91; 95% CI: 0.85–0.98; *p* = 0.009) remained significantly associated with a lower risk of acute pancreatitis (Figure 3B). However, no significant associations were observed between any essential minerals and the risk of chronic pancreatitis or other pancreatic diseases after multivariate adjustment (Figure 3C).

### 3.4 Dose–response relationships between essential minerals and pancreatic disease risk

**Figure 4:**
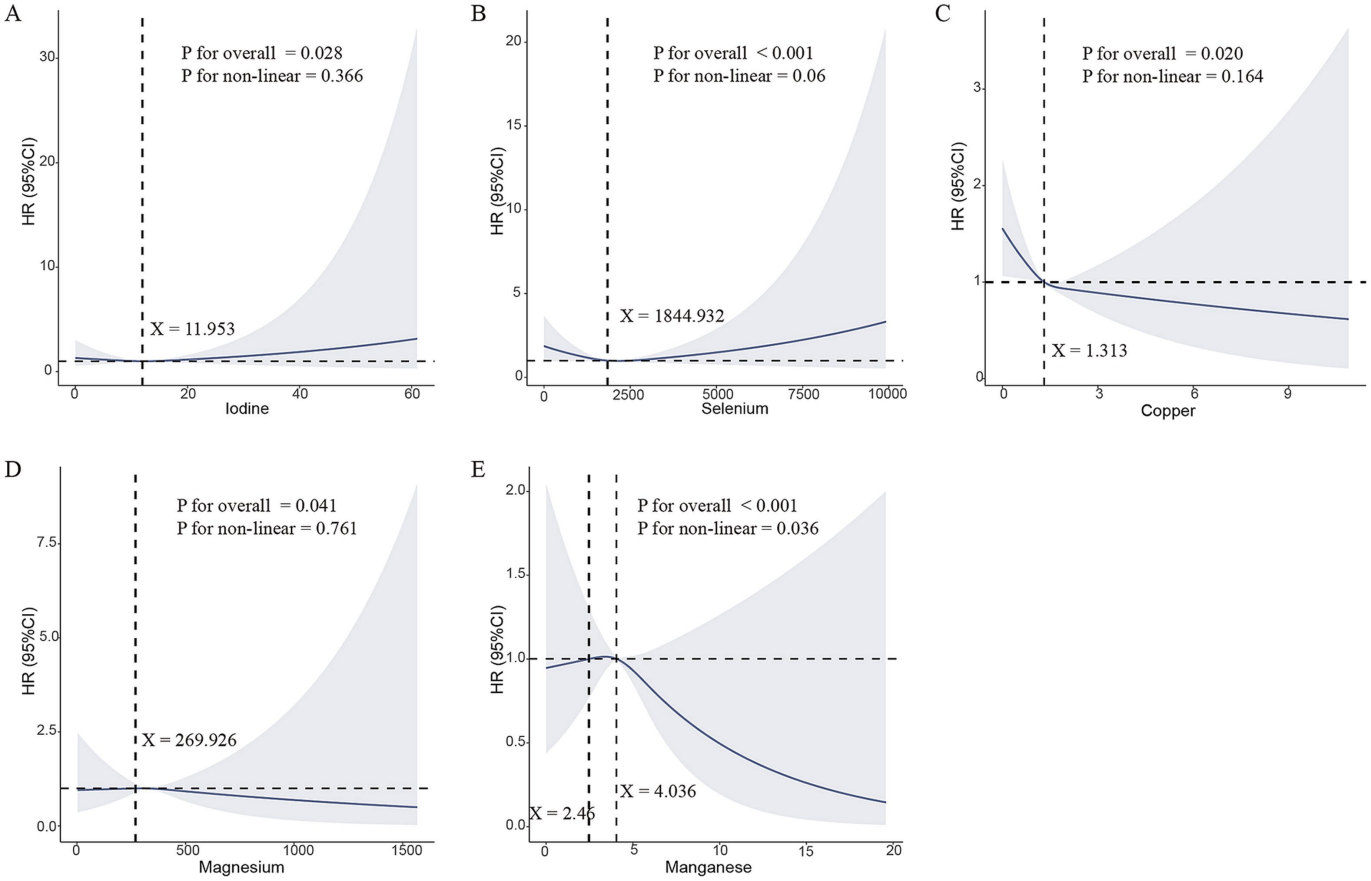
Dose–response associations between essential minerals and the risk of pancreatic diseases assessed using restricted cubic spline (RCS) regression. **(A)** Iodine and pancreatic cancer, **(B)** Selenium and pancreatic cancer, **(C)** Copper and acute pancreatitis, **(D)** Magnesium and acute pancreatitis, **(E)** Manganese and acute pancreatitis. HR: hazard ratio; CI: confidence interval. Model 2 was adjusted for age, sex, ethnicity, body mass index (BMI), Townsend Deprivation Index (TDI), annual household income before tax, Metabolic Equivalent of Task (MET), smoking status, alcohol status, diabetes, and hyperlipidemia.

A significant overall association was observed between iodine levels and pancreatic cancer risk (*p* < 0.001) with a near-significant non-linear trend (*p* for non-linearity = 0.060), suggesting a continuous increase in risk with increasing iodine concentrations. Selenium levels were also positively associated with pancreatic cancer risk (overall *p* = 0.028); however, no evidence of a non-linear relationship was observed (*p* for non-linearity = 0.366) (Figure 4). Regarding acute pancreatitis, higher copper (overall *p* = 0.020) and magnesium (overall *p* = 0.041) levels were significantly associated with a decreased disease risk, displaying linear dose–response patterns. That is, the risk of disease decreased progressively with higher circulating concentrations of these minerals. Notably, manganese showed both a significant overall association (*p* < 0.001) and a significant non-linear relationship (P for non-linearity = 0.036), indicating a potential threshold or U-shaped effect in its protective role against acute pancreatitis (Figure 4).

### 3.5 Sensitivity analyses

A series of sensitivity analyses were conducted to assess the robustness of the primary findings. These included (1) exclusion of participants with less than 2 years of follow-up, (2) exclusion of individuals with any missing covariate data, (3) mean imputation, and (4) multiple imputation for missing data, all within multivariable-adjusted Cox regression frameworks. The results of the sensitivity analyses were consistent with those of the main analysis, supporting the stability of the observed associations. Notably, the positive association between elevated iodine and selenium levels and the risk of pancreatic cancer remained significant across all sensitivity models (*p* < 0.05; Supplementary Tables S2–S5). Similarly, the protective associations of copper, magnesium, and manganese with acute pancreatitis were consistently observed in multiple sensitivity scenarios (*p* < 0.05). For instance, when excluding participants with less than 2 years of follow-up, copper continued to show a protective trend in the analysis (HR = 0.94, *p* = 0.161). In contrast, magnesium and manganese retained significant inverse associations with acute pancreatitis risk in models using multiple imputations (*p* < 0.05).

### 3.6 Subgroup analyses

Subgroup analysis revealed that the positive associations of iodine and selenium with pancreatic cancer were more pronounced among females, older individuals (≥60 years), and those with a normal BMI (*p* < 0.05), whereas such associations were not significant among males, individuals younger than 60 years, or those with obesity (*p* > 0.05; Supplementary Tables S6–S8). In contrast, the protective effects of the metal elements (copper, magnesium, and manganese) on acute pancreatitis were stronger in males and participants with normal BMI (*p* < 0.05; Supplementary Tables S6–S8). Furthermore, heterogeneity was observed across subgroups defined by smoking and alcohol status. Among current smokers and drinkers, iodine and selenium were more strongly associated with increased pancreatic cancer risk, whereas copper, magnesium, and manganese showed stronger protective effects against acute pancreatitis in former smokers (*p* < 0.05; Supplementary Tables S9–S11). Finally, stratified analyses by diabetes and dyslipidaemia status demonstrated that both the risk-enhancing effects of iodine and selenium and the protective effects of copper, magnesium, and manganese were more evident among participants without these metabolic comorbidities. These results suggest that the associations between essential minerals and pancreatic diseases may be influenced by individual metabolic and disease statuses (p < 0.05; Supplementary Tables S12, S13).

## 4 Discussion

This study demonstrates the significant differential effects of various minerals on pancreatic disorders, with elevated iodine and selenium levels exhibiting positive correlations with pancreatic cancer risk, whereas copper, magnesium, and manganese demonstrated protective effects against acute pancreatitis. PheWAS analyses further revealed extensive associations between essential minerals and multisystem disorders, particularly cardiovascular, respiratory, digestive, and metabolic diseases, suggesting that mineral dysregulation may be a common pathophysiological basis for systemic disorders. In both the unadjusted and multivariate-adjusted models, iodine and selenium consistently showed significant positive associations with the risk of pancreatic cancer. RCS analyses further identified a non-linear dose–response relationship between elevated iodine levels and pancreatic cancer risk. This finding presents an intriguing paradox, given previous reports of the protective effects of iodine against thyroid and breast cancers (16, 17), whereas both iodine deficiency and excess have been implicated in gastric carcinogenesis (18), indicating organ-specific and dose-dependent heterogeneity. Similarly, selenium, traditionally considered an anticarcinogenic element, exhibited complex biological behaviours. Selenium supplementation increases the risk of aggressive prostate cancer (19), potentially attributable to tumor-specific selenium accumulation facilitating reactive oxygen species (ROS) scavenging and subsequent proliferation (20, 21). These paradoxical findings highlight the dual biological effects of selenium in different organ systems.

Conversely, after multivariate adjustment, copper, magnesium, and manganese levels were significantly and inversely associated with the risk of acute pancreatitis. RCS analyses delineated linear protective trends for copper and magnesium, in contrast to non-linear patterns for manganese, suggesting more complex biological interactions. Mechanistically, copper and manganese are essential cofactors for antioxidant enzymes and critically regulate oxidative homeostasis. Copper serves as the catalytic centre for superoxide dismutase 1 and cytochrome c oxidase (22), whereas manganese is the active site for mitochondrial superoxide dismutase 2 (23). These metalloenzymes effectively neutralise ROS such as O₂^−^ and H₂O₂ through catalytic dismutation (24, 25), thereby mitigating oxidative damage to biomolecules. The protective mechanisms of magnesium primarily involve calcium signalling modulation and membrane stabilisation through competitive inhibition of voltage-gated calcium channels and NMDA receptors (26), coupled with the attenuation of nuclear factor kappa B- and interleukin-6-mediated inflammatory responses by suppressing lipid peroxidation (27, 28). The observed non-linear effects of manganese warrant particular attention, suggesting intricate dose–response relationships that require further mechanistic exploration.

Notably, mineral–chronic pancreatitis associations appeared to be attenuated, with potassium showing a transient positive correlation in the unadjusted models that dissipated after multivariate adjustment. This finding may reflect the predominant etiological role of chronic alcohol exposure and genetic factors in chronic pancreatitis pathogenesis (29, 30), wherein mineral metabolism plays a comparatively minor role. The long-term progressive nature of chronic pancreatitis likely requires extended follow-ups and precise exposure assessments to elucidate potential nutritional associations.

Subgroup analyses revealed significant population heterogeneity in mineral–pancreatic disease associations. The carcinogenic effects of iodine and selenium exhibited stronger associations in females, individuals older than 65 years, and normal BMI subgroups. Mechanistic interpretations include androgen-mediated downregulation of selenoprotein P transcription (31), age-related ROS accumulation (32), and obesity-induced chronic inflammation, which impairs micronutrient metabolism (33). Pronounced carcinogenic effects in smokers and alcohol consumers may stem from ROS overproduction via synergistic interactions between iodine metabolism and nicotine-induced CYP450 activation (34). In contrast, the protective effects of copper, magnesium, and manganese against acute pancreatitis were more pronounced in males and individuals with normal BMI, which was potentially mediated by androgen-regulated metallothionein expression (35, 36) and obesity-associated chronic inflammation (33).

There are significant differences between the biological effects of trace elements in organisms and the disease states observed in clinical practice. Selenium is generally regarded as an anti-cancer element, yet our research has found that it promotes the occurrence of pancreatic cancer. These differences arise from the complex interplay of multiple factors, including element form, dosage, individual genetic background, pathological status, and interactions among elements (37). Dose and chemical form are key determinants, as trace elements exhibit a characteristic biphasic “dose–response” relationship: they are essential at physiological concentrations but can become toxic at excessive levels (38). And individual genetic background and disease status can alter element metabolism, leading to varying effects of the same element across different populations (39). Additionally, interactions between elements, low bioavailability, and limitations of research models (cellular or animal versus human) complicate the direct extrapolation of biological effects observed *in vitro* to clinical outcomes (40, 41). Future research should comprehensively consider elemental forms, individual metabolic characteristics, and the disease microenvironment to more accurately elucidate the mechanisms by which trace elements influence the onset and progression of pancreatic diseases.

The results of this study substantially advance the understanding of the mineral-mediated pathophysiological mechanisms and provide novel insights into potential nutritional interventions. However, this study had several limitations. First, the generalisability of the findings may be limited by the population structure of the UK Biobank, which predominantly includes individuals of European ancestry and those with relatively high health awareness, potentially introducing selection bias. Second, the mineral intake assessment was based on repeated 24-h dietary recall questionnaires, which are inherently subject to recall bias and reporting inaccuracies and may not accurately capture long-term habitual dietary patterns. Moreover, dietary intake data do not reflect circulating mineral levels, which limits the ability to evaluate the internal exposure status. Third, although multiple potential confounders were adjusted, the observational nature of the study could not eliminate the possibility of residual confounding. Finally, the identification of pancreatic diseases relied on electronic health records, which may have been subject to misclassification or underdiagnosis, particularly for subclinical cases or those with variable diagnostic criteria.

## 5 Conclusion

This comprehensive investigation established essential minerals as significant modulators of pancreatic disease risk with multisystem implications. Differential associations (iodine/selenium carcinogenicity vs. copper/magnesium/manganese protection) and population heterogeneity provide crucial insights for etiological research and precise nutritional strategies. These findings provide novel insights into the metabolic aetiologies of pancreatic disorders and provide evidence-based nutritional intervention strategies. However, clinical translation requires further validation through mechanistic studies and randomised controlled trials.

## Data Availability

The original contributions presented in the study are included in the article/Supplementary material, further inquiries can be directed to the corresponding authors.
