# Supplementary material for: Essential minerals and risk of pancreatic diseases: a large-scale prospective cohort study
Source: Front Nutr. 2026 Apr 29;13:1773339. doi: 10.3389/fnut.2026.1773339 (PMC13174579; doi:10.3389/fnut.2026.1773339)
Supplement: Supplementary file 1 [file Table_1.docx]

Essential Minerals and the Risk of Pancreatic Diseases: A Large-Scale Prospective Cohort Study

CONTENTS

[Table S1. The association between essential minerals and the risk of pancreatic diseases. 2](#_Toc199926366)

[Table S2. Sensitivity analysis between essential minerals and the risk of pancreatic diseases with participants excluding follow-up within 2 years. 3](#_Toc199926367)

[Table S3. Sensitivity analysis of the association between essential minerals and the risk of pancreatic diseases excluding participants with any missing variate data. 4](#_Toc199926368)

[Table S4. Sensitivity analysis of the association between essential minerals and pancreatic disease risk after mean imputation for variables. 5](#_Toc199926369)

[Table S5. Sensitivity analysis of the association between essential minerals and pancreatic disease risk after multiple imputation for variables. 6](#_Toc199926370)

[Table S6. Stratified analysis of the association between essential minerals and pancreatic disease risk by sex. 7](#_Toc199926371)

[Table S7. Stratified analysis of the association between essential minerals and pancreatic disease risk by age. 8](#_Toc199926372)

[Table S8. Stratified analysis of the association between essential minerals and pancreatic disease risk by body mass index. 9](#_Toc199926373)

[Table S9. Stratified analysis of the association between essential minerals and pancreatic cancer risk by smoking status. 10](#_Toc199926374)

[Table S 10. Stratified analysis of the association between essential minerals and acute pancreatitis risk by smoking status. 11](#_Toc199926375)

[Table S11. Stratified analysis of the association between essential minerals and acute risk of chronic pancreatitis and others by smoking status. 12](#_Toc199926376)

[Table S12. Stratified analysis of the association between essential minerals and pancreatic disease risk by diabetes disease. 13](#_Toc199926377)

[Table S13. Stratified analysis of the association between essential minerals and pancreatic disease risk by dyslipidemia disease. 14](#_Toc199926378)

# Table S1. The association between essential minerals and the risk of pancreatic diseases.

| **Variable** | **Pancreatic Cancer** | | **Acute Pancreatitis** | | **Chronic Pancreatitis and others** | |
| --- | --- | --- | --- | --- | --- | --- |
|  | **HR (95%CI)** | **P_value** | **HR (95%CI)** | **P_value** | **HR (95%CI)** | **P_value** |
| Calcium | 1.01 (0.89, 1.14) | 0.896 | 0.95 (0.89, 1.02) | 0.161 | 1.01 (0.95, 1.08) | 0.668 |
| Chloride | 0.99 (0.87, 1.12) | 0.821 | 1.05 (0.98, 1.11) | 0.140 | 1.00 (0.94, 1.07) | 0.885 |
| Copper | 1.05 (0.94, 1.18) | 0.362 | 0.91 (0.85, 0.98) | 0.008 | 1.06 (1.00, 1.12) | 0.054 |
| Iodine | 1.17 (1.07, 1.28) | 0.000 | 1.02 (0.96, 1.09) | 0.445 | 1.04 (0.98, 1.11) | 0.171 |
| Iron | 1.04 (0.93, 1.18) | 0.483 | 0.93 (0.87, 1.00) | 0.040 | 1.04 (0.98, 1.10) | 0.249 |
| Magnesium | 0.99 (0.88, 1.12) | 0.901 | 0.91 (0.85, 0.97) | 0.004 | 1.02 (0.95, 1.08) | 0.624 |
| Manganese | 0.92 (0.81, 1.05) | 0.227 | 0.88 (0.82, 0.94) | <0.001 | 1.00 (0.94, 1.06) | 0.985 |
| Phosphorus | 1.02 (0.90, 1.15) | 0.749 | 0.98 (0.92, 1.05) | 0.595 | 1.03 (0.97, 1.09) | 0.395 |
| Potassium | 1.04 (0.92, 1.17) | 0.534 | 0.99 (0.92, 1.05) | 0.674 | 1.08 (1.02, 1.15) | 0.007 |
| Selenium | 1.12 (1.01, 1.24) | 0.028 | 1.04 (0.98, 1.11) | 0.197 | 1.00 (0.94, 1.07) | 0.907 |
| Sodium | 0.99 (0.87, 1.12) | 0.827 | 1.05 (0.99, 1.12) | 0.089 | 1.01 (0.94, 1.07) | 0.870 |
| Zinc | 1.06 (0.95, 1.20) | 0.302 | 0.98 (0.92, 1.05) | 0.561 | 1.04 (0.98, 1.10) | 0.244 |

Model 1: Unadjusted model without covariate adjustment. HR: hazard ratio; CI: confidence interval.

# Table S2. Sensitivity analysis between essential minerals and the risk of pancreatic diseases with participants excluding follow-up within 2 years.

| **Variable** | **Pancreatic Cancer** | | **Acute Pancreatitis** | | **Chronic Pancreatitis and others** | |
| --- | --- | --- | --- | --- | --- | --- |
|  | **HR (95%CI)** | **P_value** | **HR (95%CI)** | **P_value** | **HR (95%CI)** | **P_value** |
| Calcium | 0.98 (0.86, 1.12) | 0.817 | 0.95 (0.89, 1.01) | 0.112 | 1.00 (0.94, 1.07) | 0.883 |
| Chloride | 0.97 (0.85, 1.11) | 0.689 | 1.00 (0.94, 1.07) | 0.904 | 1.00 (0.93, 1.06) | 0.903 |
| Copper | 1.02 (0.91, 1.15) | 0.702 | 0.92 (0.86, 0.98) | 0.015 | 1.04 (0.99, 1.11) | 0.141 |
| Iodine | 1.14 (1.03, 1.25) | 0.008 | 1.02 (0.95, 1.08) | 0.617 | 1.01 (0.95, 1.07) | 0.760 |
| Iron | 1.01 (0.89, 1.15) | 0.841 | 0.94 (0.88, 1.00) | 0.063 | 1.02 (0.96, 1.09) | 0.455 |
| Magnesium | 0.97 (0.85, 1.11) | 0.632 | 0.92 (0.86, 0.99) | 0.017 | 1.01 (0.95, 1.08) | 0.706 |
| Manganese | 0.91 (0.79, 1.04) | 0.153 | 0.91 (0.85, 0.98) | 0.009 | 1.00 (0.93, 1.06) | 0.922 |
| Phosphorus | 1.00 (0.88, 1.14) | 1.000 | 0.97 (0.91, 1.03) | 0.337 | 1.02 (0.96, 1.09) | 0.547 |
| Potassium | 0.97 (0.85, 1.10) | 0.646 | 0.98 (0.92, 1.04) | 0.491 | 1.04 (0.98, 1.11) | 0.189 |
| Selenium | 1.13 (1.02, 1.26) | 0.025 | 1.04 (0.98, 1.11) | 0.208 | 1.02 (0.95, 1.08) | 0.590 |
| Sodium | 0.98 (0.86, 1.12) | 0.732 | 1.01 (0.94, 1.07) | 0.847 | 1.00 (0.93, 1.06) | 0.932 |
| Zinc | 1.05 (0.92, 1.18) | 0.478 | 0.96 (0.90, 1.02) | 0.187 | 1.03 (0.97, 1.10) | 0.332 |

HR: hazard ratio; CI: confidence interval. Model 2 were further adjusted with age, sex, ethnicity, body mass index (BMI), Townsend deprivation index (TDI), annual household income before tax, Metabolic Equivalent of Task (MET), smoking status, alcohol status, diabetes and dyslipidemia diseases.

# Table S3. Sensitivity analysis of the association between essential minerals and the risk of pancreatic diseases excluding participants with any missing variate data.

| **Variable** | **Pancreatic Cancer** | | **Acute Pancreatitis** | | **Chronic Pancreatitis and others** | |
| --- | --- | --- | --- | --- | --- | --- |
|  | **HR (95%CI)** | **P_value** | **HR (95%CI)** | **P_value** | **HR (95%CI)** | **P_value** |
| Calcium | 1.07 (0.92, 1.24) | 0.405 | 0.94 (0.87, 1.02) | 0.142 | 1.02 (0.94, 1.10) | 0.645 |
| Chloride | 1.06 (0.91, 1.23) | 0.440 | 1.01 (0.93, 1.09) | 0.856 | 1.01 (0.94, 1.10) | 0.732 |
| Copper | 1.09 (0.96, 1.23) | 0.202 | 0.92 (0.84, 1.00) | 0.050 | 1.05 (0.98, 1.13) | 0.180 |
| Iodine | 1.21 (1.12, 1.31) | <0.001 | 1.03 (0.95, 1.11) | 0.509 | 1.02 (0.95, 1.10) | 0.596 |
| Iron | 1.12 (0.97, 1.30) | 0.120 | 0.93 (0.85, 1.00) | 0.063 | 1.03 (0.95, 1.11) | 0.519 |
| Magnesium | 1.04 (0.89, 1.21) | 0.619 | 0.92 (0.85, 1.00) | 0.049 | 1.01 (0.94, 1.10) | 0.743 |
| Manganese | 0.91 (0.77, 1.07) | 0.246 | 0.91 (0.84, 0.99) | 0.031 | 0.99 (0.92, 1.08) | 0.888 |
| Phosphorus | 1.11 (0.97, 1.29) | 0.140 | 0.97 (0.89, 1.05) | 0.384 | 1.02 (0.94, 1.10) | 0.692 |
| Potassium | 1.07 (0.92, 1.23) | 0.386 | 0.97 (0.90, 1.05) | 0.518 | 1.06 (0.98, 1.14) | 0.120 |
| Selenium | 1.23 (1.10, 1.37) | 0.000 | 1.04 (0.96, 1.12) | 0.357 | 1.01 (0.93, 1.09) | 0.809 |
| Sodium | 1.07 (0.93, 1.24) | 0.335 | 1.01 (0.94, 1.10) | 0.733 | 1.01 (0.93, 1.09) | 0.866 |
| Zinc | 1.14 (0.99, 1.31) | 0.072 | 0.94 (0.87, 1.02) | 0.155 | 0.99 (0.92, 1.08) | 0.886 |

HR: hazard ratio; CI: confidence interval. Model 2 were further adjusted with age, sex, ethnicity, body mass index (BMI), Townsend deprivation index (TDI), annual household income before tax, Metabolic Equivalent of Task (MET), smoking status, alcohol status, diabetes and dyslipidemia diseases.

# Table S4. Sensitivity analysis of the association between essential minerals and pancreatic disease risk after mean imputation for variables.

| **Variable** | **Pancreatic Cancer** | | **Acute Pancreatitis** | | **Chronic Pancreatitis and others** | |
| --- | --- | --- | --- | --- | --- | --- |
|  | **HR (95%CI)** | **P_value** | **HR (95%CI)** | **P_value** | **HR (95%CI)** | **P_value** |
| Calcium | 0.99 (0.87, 1.13) | 0.879 | 1.00 (1.00, 1.00) | 0.130 | 1.00 (1.00, 1.00) | 0.913 |
| Chloride | 1.00 (0.87, 1.14) | 0.973 | 1.00 (1.00, 1.00) | 0.698 | 1.00 (1.00, 1.00) | 0.719 |
| Copper | 1.03 (0.91, 1.17) | 0.596 | 0.87 (0.78, 0.99) | 0.029 | 1.07 (0.96, 1.19) | 0.205 |
| Iodine | 1.14 (1.03, 1.26) | 0.011 | 1.00 (1.00, 1.00) | 0.732 | 1.00 (1.00, 1.00) | 0.707 |
| Iron | 1.03 (0.90, 1.17) | 0.703 | 0.99 (0.97, 1.00) | 0.102 | 1.01 (0.99, 1.02) | 0.492 |
| Magnesium | 0.97 (0.84, 1.11) | 0.637 | 1.00 (1.00, 1.00) | 0.031 | 1.00 (1.00, 1.00) | 0.845 |
| Manganese | 0.93 (0.81, 1.07) | 0.298 | 0.95 (0.91, 0.99) | 0.017 | 1.00 (0.96, 1.04) | 0.893 |
| Phosphorus | 1.01 (0.89, 1.15) | 0.864 | 1.00 (1.00, 1.00) | 0.393 | 1.00 (1.00, 1.00) | 0.716 |
| Potassium | 0.98 (0.86, 1.12) | 0.740 | 1.00 (1.00, 1.00) | 0.513 | 1.00 (1.00, 1.00) | 0.157 |
| Selenium | 1.14 (1.03, 1.28) | 0.015 | 1.00 (1.00, 1.00) | 0.189 | 1.00 (1.00, 1.00) | 0.764 |
| Sodium | 1.00 (0.88, 1.15) | 0.976 | 1.00 (1.00, 1.00) | 0.649 | 1.00 (1.00, 1.00) | 0.747 |
| Zinc | 1.06 (0.93, 1.20) | 0.381 | 0.99 (0.97, 1.01) | 0.243 | 1.01 (0.99, 1.02) | 0.550 |

HR: hazard ratio; CI: confidence interval. Model 2 were further adjusted with age, sex, ethnicity, body mass index (BMI), Townsend deprivation index (TDI), annual household income before tax, Metabolic Equivalent of Task (MET), smoking status, alcohol status, diabetes and dyslipidemia diseases.

# Table S5. Sensitivity analysis of the association between essential minerals and pancreatic disease risk after multiple imputation for variables.

| **Variable** | **Pancreatic Cancer** | | **Acute Pancreatitis** | | **Chronic Pancreatitis and others** | |
| --- | --- | --- | --- | --- | --- | --- |
|  | **HR (95%CI)** | **P_value** | **HR (95%CI)** | **P_value** | **HR (95%CI)** | **P_value** |
| Calcium | 1.00 (1.00, 1.00) | 0.109 | 1.00 (1.00, 1.00) | 0.043 | 1.00 (1.00, 1.00) | 0.882 |
| Chloride | 1.00 (1.00, 1.00) | 0.355 | 1.00 (1.00, 1.00) | 0.550 | 1.00 (1.00, 1.00) | 0.983 |
| Copper | 1.13 (0.97, 1.31) | 0.109 | 0.94 (0.87, 1.02) | 0.161 | 1.07 (0.99, 1.16) | 0.076 |
| Iodine | 1.00 (1.00, 1.00) | 0.012 | 1.00 (1.00, 1.00) | 0.457 | 1.00 (1.00, 1.00) | 0.284 |
| Iron | 1.02 (0.99, 1.04) | 0.143 | 0.99 (0.97, 1.00) | 0.010 | 1.00 (0.99, 1.02) | 0.489 |
| Magnesium | 1.00 (1.00, 1.00) | 0.105 | 1.00 (1.00, 1.00) | 0.017 | 1.00 (1.00, 1.00) | 0.602 |
| Manganese | 1.02 (0.97, 1.07) | 0.490 | 0.97 (0.94, 1.00) | 0.022 | 1.01 (0.98, 1.03) | 0.651 |
| Phosphorus | 1.00 (1.00, 1.00) | 0.055 | 1.00 (1.00, 1.00) | 0.162 | 1.00 (1.00, 1.00) | 0.987 |
| Potassium | 1.00 (1.00, 1.00) | 0.364 | 1.00 (1.00, 1.00) | 0.110 | 1.00 (1.00, 1.00) | 0.420 |
| Selenium | 1.00 (1.00, 1.00) | 0.629 | 1.00 (1.00, 1.00) | 0.095 | 1.00 (1.00, 1.00) | 0.330 |
| Sodium | 1.00 (1.00, 1.00) | 0.439 | 1.00 (1.00, 1.00) | 0.467 | 1.00 (1.00, 1.00) | 0.941 |
| Zinc | 1.03 (1.01, 1.06) | 0.009 | 0.99 (0.98, 1.01) | 0.281 | 1.01 (0.99, 1.02) | 0.335 |

HR: hazard ratio; CI: confidence interval. Model 2 were further adjusted with age, sex, ethnicity, body mass index (BMI), Townsend deprivation index (TDI), annual household income before tax, Metabolic Equivalent of Task (MET), smoking status, alcohol status, diabetes and dyslipidemia diseases.

# Table S6. Stratified analysis of the association between essential minerals and pancreatic disease risk by sex.

| **Variable** | **Pancreatic Cancer** | | | | **Acute Pancreatitis** | | | | **Chronic Pancreatitis and others** | | | |
| --- | --- | --- | --- | --- | --- | --- | --- | --- | --- | --- | --- | --- |
|  | **Male** | | **Female** | | **Male** | | **Female** | | **Male** | | **Female** | |
|  | HR (95%CI) | P | HR (95%CI) | P | HR (95%CI) | P | HR (95%CI) | P | HR (95%CI) | P | HR (95%CI) | P |
| Calcium | 0.94 (0.77, 1.14) | 0.531 | 1.03 (0.87, 1.22) | 0.718 | 0.90 (0.81, 0.99) | 0.037 | 0.99 (0.91, 1.08) | 0.813 | 0.99 (0.90, 1.09) | 0.789 | 1.00 (0.92, 1.09) | 0.951 |
| Chloride | 0.87 (0.70, 1.08) | 0.199 | 1.05 (0.89, 1.23) | 0.569 | 0.96 (0.87, 1.06) | 0.444 | 1.05 (0.97, 1.13) | 0.276 | 1.02 (0.92, 1.12) | 0.747 | 0.97 (0.89, 1.06) | 0.504 |
| Copper | 0.99 (0.82, 1.19) | 0.893 | 1.05 (0.91, 1.22) | 0.515 | 0.91 (0.83, 1.01) | 0.087 | 0.92 (0.84, 1.01) | 0.097 | 1.08 (0.99, 1.17) | 0.081 | 1.01 (0.93, 1.09) | 0.869 |
| Iodine | 0.95 (0.78, 1.16) | 0.607 | 1.20 (1.10, 1.30) | 0.000 | 0.99 (0.90, 1.09) | 0.780 | 1.03 (0.95, 1.12) | 0.474 | 1.05 (0.96, 1.15) | 0.269 | 0.97 (0.89, 1.06) | 0.523 |
| Iron | 1.01 (0.83, 1.22) | 0.959 | 1.03 (0.87, 1.21) | 0.748 | 0.91 (0.82, 1.00) | 0.061 | 0.96 (0.88, 1.05) | 0.410 | 1.06 (0.96, 1.16) | 0.247 | 0.99 (0.91, 1.08) | 0.880 |
| Magnesium | 0.94 (0.76, 1.14) | 0.516 | 1.00 (0.84, 1.18) | 0.971 | 0.85 (0.77, 0.94) | 0.002 | 0.98 (0.90, 1.07) | 0.627 | 1.04 (0.95, 1.14) | 0.405 | 0.98 (0.90, 1.06) | 0.604 |
| Manganese | 0.89 (0.73, 1.09) | 0.274 | 0.92 (0.77, 1.09) | 0.332 | 0.86 (0.77, 0.95) | 0.003 | 0.96 (0.88, 1.05) | 0.404 | 1.02 (0.93, 1.12) | 0.693 | 0.98 (0.90, 1.07) | 0.613 |
| Phosphorus | 0.91 (0.75, 1.12) | 0.373 | 1.07 (0.91, 1.26) | 0.425 | 0.91 (0.82, 1.00) | 0.055 | 1.02 (0.94, 1.10) | 0.691 | 1.04 (0.95, 1.15) | 0.389 | 0.99 (0.91, 1.07) | 0.754 |
| Potassium | 0.91 (0.75, 1.11) | 0.358 | 1.03 (0.87, 1.21) | 0.746 | 0.89 (0.81, 0.98) | 0.017 | 1.05 (0.97, 1.14) | 0.221 | 1.09 (1.00, 1.19) | 0.054 | 1.00 (0.92, 1.08) | 0.913 |
| Selenium | 1.05 (0.88, 1.26) | 0.572 | 1.18 (1.04, 1.35) | 0.013 | 1.01 (0.92, 1.11) | 0.821 | 1.06 (0.98, 1.15) | 0.118 | 1.11 (1.02, 1.20) | 0.019 | 0.93 (0.84, 1.01) | 0.097 |
| Sodium | 0.86 (0.69, 1.07) | 0.176 | 1.06 (0.90, 1.24) | 0.488 | 0.98 (0.89, 1.09) | 0.735 | 1.04 (0.95, 1.12) | 0.402 | 1.01 (0.91, 1.12) | 0.864 | 0.98 (0.90, 1.07) | 0.652 |
| Zinc | 1.00 (0.83, 1.22) | 0.976 | 1.07 (0.92, 1.26) | 0.375 | 0.91 (0.83, 1.01) | 0.076 | 1.00 (0.92, 1.08) | 0.919 | 1.03 (0.93, 1.13) | 0.568 | 1.01 (0.93, 1.10) | 0.787 |

HR: hazard ratio; CI: confidence interval. Model 2 were further adjusted with age, ethnicity, body mass index (BMI), Townsend deprivation index (TDI), annual household income before tax, Metabolic Equivalent of Task (MET), smoking status, alcohol status, diabetes and dyslipidemia diseases.

# Table S7. Stratified analysis of the association between essential minerals and pancreatic disease risk by age.

| **Variable** | **Pancreatic Cancer** | | | | **Acute Pancreatitis** | | | | **Chronic Pancreatitis and others** | | | |
| --- | --- | --- | --- | --- | --- | --- | --- | --- | --- | --- | --- | --- |
|  | **< 60 y** | | **≥ 60 y** | | **< 60 y** | | **≥ 60 y** | | **< 60 y** | | **≥ 60 y** | |
|  | HR (95%CI) | P | HR (95%CI) | P | HR (95%CI) | P | HR (95%CI) | P | HR (95%CI) | P | HR (95%CI) | P |
| Calcium | 0.92 (0.74, 1.15) | 0.480 | 1.02 (0.87, 1.19) | 0.811 | 0.92 (0.84, 1.01) | 0.069 | 0.98 (0.89, 1.08) | 0.666 | 0.94 (0.85, 1.05) | 0.273 | 1.03 (0.95, 1.12) | 0.487 |
| Chloride | 0.90 (0.72, 1.13) | 0.362 | 1.00 (0.85, 1.17) | 0.989 | 0.97 (0.89, 1.06) | 0.495 | 1.05 (0.96, 1.15) | 0.308 | 0.92 (0.83, 1.02) | 0.120 | 1.02 (0.94, 1.11) | 0.610 |
| Copper | 0.98 (0.79, 1.22) | 0.883 | 1.05 (0.91, 1.21) | 0.512 | 0.93 (0.84, 1.02) | 0.118 | 0.92 (0.84, 1.02) | 0.099 | 0.99 (0.89, 1.09) | 0.777 | 1.07 (1.00, 1.15) | 0.053 |
| Iodine | 1.06 (0.87, 1.29) | 0.581 | 1.16 (1.06, 1.28) | 0.002 | 0.98 (0.89, 1.07) | 0.672 | 1.05 (0.96, 1.14) | 0.285 | 1.04 (0.95, 1.15) | 0.389 | 1.00 (0.92, 1.08) | 0.929 |
| Iron | 0.85 (0.68, 1.06) | 0.153 | 1.12 (0.96, 1.30) | 0.145 | 0.91 (0.83, 1.00) | 0.050 | 0.97 (0.88, 1.07) | 0.549 | 0.95 (0.86, 1.05) | 0.338 | 1.07 (0.99, 1.16) | 0.103 |
| Magnesium | 0.86 (0.69, 1.08) | 0.206 | 1.03 (0.88, 1.20) | 0.706 | 0.91 (0.83, 0.99) | 0.037 | 0.94 (0.85, 1.04) | 0.213 | 0.97 (0.88, 1.07) | 0.555 | 1.03 (0.95, 1.12) | 0.451 |
| Manganese | 0.78 (0.61, 0.99) | 0.039 | 0.98 (0.84, 1.15) | 0.840 | 0.93 (0.85, 1.02) | 0.118 | 0.91 (0.82, 1.00) | 0.047 | 0.97 (0.87, 1.07) | 0.554 | 1.02 (0.94, 1.11) | 0.622 |
| Phosphorus | 0.87 (0.69, 1.08) | 0.208 | 1.07 (0.92, 1.24) | 0.406 | 0.96 (0.88, 1.05) | 0.374 | 0.97 (0.89, 1.07) | 0.583 | 0.97 (0.88, 1.08) | 0.594 | 1.03 (0.95, 1.12) | 0.449 |
| Potassium | 0.95 (0.77, 1.18) | 0.650 | 1.00 (0.86, 1.17) | 0.959 | 0.96 (0.87, 1.05) | 0.333 | 1.01 (0.92, 1.10) | 0.876 | 1.02 (0.92, 1.12) | 0.716 | 1.07 (0.99, 1.15) | 0.110 |
| Selenium | 1.03 (0.85, 1.26) | 0.734 | 1.17 (1.03, 1.34) | 0.017 | 1.04 (0.96, 1.13) | 0.379 | 1.05 (0.96, 1.14) | 0.325 | 1.04 (0.95, 1.14) | 0.370 | 0.98 (0.90, 1.07) | 0.660 |
| Sodium | 0.86 (0.69, 1.08) | 0.198 | 1.03 (0.88, 1.20) | 0.729 | 0.97 (0.89, 1.06) | 0.501 | 1.05 (0.96, 1.15) | 0.277 | 0.90 (0.81, 1.00) | 0.052 | 1.04 (0.96, 1.12) | 0.399 |
| Zinc | 0.78 (0.62, 0.99) | 0.043 | 1.17 (1.02, 1.35) | 0.022 | 0.94 (0.86, 1.03) | 0.207 | 0.97 (0.89, 1.07) | 0.577 | 0.96 (0.86, 1.06) | 0.408 | 1.05 (0.97, 1.14) | 0.191 |

HR: hazard ratio; CI: confidence interval. Model 2 were further adjusted with sex, ethnicity, body mass index (BMI), Townsend deprivation index (TDI), annual household income before tax, Metabolic Equivalent of Task (MET), smoking status, alcohol status, diabetes and dyslipidemia diseases.

# Table S8. Stratified analysis of the association between essential minerals and pancreatic disease risk by body mass index.

| **Variable** | **Pancreatic Cancer** | | | | **Acute Pancreatitis** | | | | **Chronic Pancreatitis and others** | | | |
| --- | --- | --- | --- | --- | --- | --- | --- | --- | --- | --- | --- | --- |
|  | **< 30 kg/m^2^** | | **≥ 30 kg/m^2^** | | **< 30 kg/m^2^** | | **≥ 30 kg/m^2^** | | **< 30 kg/m^2^** | | **≥ 30 kg/m^2^** | |
|  | HR (95%CI) | P | HR (95%CI) | P | HR (95%CI) | P | HR (95%CI) | P | HR (95%CI) | P | HR (95%CI) | P |
| Calcium | 0.97 (0.83, 1.13) | 0.671 | 1.05 (0.82, 1.33) | 0.713 | 0.93 (0.85, 1.01) | 0.094 | 0.98 (0.88, 1.08) | 0.663 | 1.02 (0.95, 1.10) | 0.594 | 0.95 (0.84, 1.07) | 0.366 |
| Chloride | 0.97 (0.83, 1.13) | 0.687 | 1.01 (0.80, 1.27) | 0.951 | 1.01 (0.93, 1.10) | 0.727 | 1.03 (0.93, 1.13) | 0.581 | 1.00 (0.93, 1.08) | 0.929 | 0.97 (0.86, 1.08) | 0.546 |
| Copper | 1.03 (0.89, 1.18) | 0.706 | 1.02 (0.81, 1.28) | 0.874 | 0.89 (0.82, 0.98) | 0.015 | 0.95 (0.85, 1.05) | 0.308 | 1.06 (0.99, 1.14) | 0.074 | 0.99 (0.88, 1.11) | 0.866 |
| Iodine | 1.15 (1.04, 1.27) | 0.008 | 1.08 (0.87, 1.34) | 0.474 | 1.02 (0.95, 1.11) | 0.564 | 0.99 (0.89, 1.10) | 0.869 | 0.99 (0.92, 1.07) | 0.856 | 1.05 (0.94, 1.17) | 0.417 |
| Iron | 1.01 (0.87, 1.17) | 0.937 | 1.05 (0.83, 1.34) | 0.657 | 0.91 (0.84, 0.99) | 0.037 | 0.97 (0.87, 1.08) | 0.556 | 1.03 (0.96, 1.11) | 0.409 | 1.01 (0.90, 1.13) | 0.921 |
| Magnesium | 0.97 (0.84, 1.13) | 0.741 | 0.96 (0.74, 1.23) | 0.741 | 0.89 (0.82, 0.97) | 0.008 | 0.96 (0.86, 1.06) | 0.419 | 1.03 (0.96, 1.11) | 0.391 | 0.95 (0.85, 1.08) | 0.448 |
| Manganese | 0.92 (0.79, 1.07) | 0.298 | 0.84 (0.64, 1.10) | 0.213 | 0.88 (0.81, 0.96) | 0.003 | 0.94 (0.84, 1.05) | 0.287 | 1.01 (0.94, 1.09) | 0.728 | 0.96 (0.85, 1.09) | 0.544 |
| Phosphorus | 1.01 (0.87, 1.17) | 0.908 | 0.99 (0.78, 1.25) | 0.916 | 0.94 (0.87, 1.03) | 0.191 | 1.02 (0.92, 1.12) | 0.759 | 1.02 (0.95, 1.10) | 0.586 | 1.00 (0.89, 1.12) | 0.987 |
| Potassium | 0.97 (0.83, 1.13) | 0.673 | 1.00 (0.79, 1.26) | 0.973 | 0.96 (0.89, 1.05) | 0.386 | 0.99 (0.90, 1.10) | 0.901 | 1.07 (1.00, 1.15) | 0.049 | 0.97 (0.87, 1.09) | 0.627 |
| Selenium | 1.14 (1.01, 1.29) | 0.040 | 1.11 (0.90, 1.36) | 0.337 | 1.04 (0.96, 1.13) | 0.295 | 1.05 (0.96, 1.16) | 0.287 | 0.99 (0.92, 1.07) | 0.831 | 1.06 (0.95, 1.18) | 0.306 |
| Sodium | 0.97 (0.83, 1.14) | 0.714 | 1.01 (0.81, 1.28) | 0.901 | 1.02 (0.93, 1.10) | 0.707 | 1.04 (0.94, 1.14) | 0.477 | 1.00 (0.92, 1.08) | 0.935 | 0.98 (0.88, 1.10) | 0.787 |
| Zinc | 1.05 (0.90, 1.22) | 0.536 | 1.05 (0.84, 1.31) | 0.684 | 0.92 (0.84, 1.01) | 0.067 | 1.03 (0.94, 1.13) | 0.553 | 1.04 (0.96, 1.12) | 0.361 | 1.00 (0.89, 1.11) | 0.942 |

HR: hazard ratio; CI: confidence interval. Model 2 were further adjusted with age, sex, ethnicity, Townsend deprivation index (TDI), annual household income before tax, Metabolic Equivalent of Task (MET), smoking status, alcohol status, diabetes and dyslipidemia diseases.

# Table S9. Stratified analysis of the association between essential minerals and pancreatic cancer risk by smoking status.

| **Variable** | **Smoking status** | | | | | | **Alcohol status** | | | | | |
| --- | --- | --- | --- | --- | --- | --- | --- | --- | --- | --- | --- | --- |
|  | **Never** | | **Previous** | | **Current** | | **Never** | | **Previous** | | **Current** | |
|  | HR (95%CI) | P | HR (95%CI) | P | HR (95%CI) | P | HR (95%CI) | P | HR (95%CI) | P | HR (95%CI) | P |
| Calcium | 1.04 (0.87, 1.25) | 0.645 | 0.85 (0.69, 1.06) | 0.145 | 1.22 (0.90, 1.66) | 0.205 | 1.02 (0.59, 1.74) | 0.954 | 0.99 (0.63, 1.56) | 0.978 | 0.99 (0.86, 1.13) | 0.851 |
| Chloride | 0.99 (0.82, 1.21) | 0.946 | 0.93 (0.76, 1.14) | 0.472 | 1.09 (0.79, 1.52) | 0.598 | 0.89 (0.47, 1.70) | 0.730 | 1.08 (0.67, 1.73) | 0.753 | 0.97 (0.85, 1.12) | 0.697 |
| Copper | 1.08 (0.91, 1.27) | 0.389 | 0.96 (0.79, 1.17) | 0.677 | 1.06 (0.79, 1.43) | 0.686 | 1.09 (0.80, 1.48) | 0.598 | 0.95 (0.58, 1.56) | 0.852 | 1.02 (0.90, 1.16) | 0.709 |
| Iodine | 1.14 (1.01, 1.29) | 0.030 | 0.98 (0.81, 1.20) | 0.874 | 1.41 (1.17, 1.71) | 0.000 | 0.85 (0.43, 1.68) | 0.634 | 1.01 (0.64, 1.57) | 0.981 | 1.15 (1.04, 1.27) | 0.005 |
| Iron | 1.04 (0.87, 1.25) | 0.665 | 0.98 (0.80, 1.19) | 0.827 | 1.09 (0.78, 1.53) | 0.620 | 0.85 (0.46, 1.59) | 0.615 | 1.08 (0.70, 1.67) | 0.716 | 1.03 (0.90, 1.17) | 0.718 |
| Magnesium | 0.99 (0.82, 1.19) | 0.909 | 0.88 (0.72, 1.09) | 0.241 | 1.21 (0.87, 1.68) | 0.263 | 1.00 (0.65, 1.55) | 0.982 | 1.03 (0.67, 1.58) | 0.902 | 0.97 (0.84, 1.11) | 0.619 |
| Manganese | 1.00 (0.83, 1.21) | 0.980 | 0.81 (0.66, 1.01) | 0.058 | 0.87 (0.59, 1.28) | 0.481 | 0.98 (0.60, 1.61) | 0.935 | 0.88 (0.54, 1.46) | 0.630 | 0.90 (0.78, 1.04) | 0.166 |
| Phosphorus | 1.01 (0.84, 1.21) | 0.955 | 0.91 (0.74, 1.11) | 0.346 | 1.30 (0.96, 1.75) | 0.088 | 0.91 (0.52, 1.59) | 0.732 | 1.03 (0.68, 1.57) | 0.891 | 1.01 (0.88, 1.15) | 0.932 |
| Potassium | 0.91 (0.75, 1.10) | 0.342 | 0.95 (0.78, 1.16) | 0.616 | 1.31 (0.96, 1.80) | 0.090 | 0.90 (0.51, 1.58) | 0.715 | 0.97 (0.63, 1.51) | 0.903 | 0.98 (0.86, 1.12) | 0.799 |
| Selenium | 1.09 (0.93, 1.29) | 0.284 | 1.02 (0.84, 1.24) | 0.842 | 1.41 (1.18, 1.68) | 0.000 | 0.80 (0.39, 1.64) | 0.533 | 1.23 (0.85, 1.79) | 0.274 | 1.14 (1.01, 1.27) | 0.029 |
| Sodium | 1.02 (0.84, 1.23) | 0.875 | 0.94 (0.77, 1.15) | 0.538 | 1.03 (0.73, 1.44) | 0.875 | 0.88 (0.46, 1.71) | 0.716 | 1.05 (0.65, 1.72) | 0.830 | 0.98 (0.85, 1.12) | 0.773 |
| Zinc | 1.02 (0.85, 1.23) | 0.833 | 1.00 (0.82, 1.22) | 0.998 | 1.26 (0.94, 1.69) | 0.120 | 0.79 (0.40, 1.54) | 0.483 | 1.04 (0.69, 1.56) | 0.849 | 1.06 (0.93, 1.20) | 0.396 |

HR: hazard ratio; CI: confidence interval. Model 2 were further adjusted with age, sex, ethnicity, body mass index (BMI), Townsend deprivation index (TDI), annual household income before tax, Metabolic Equivalent of Task (MET), smoking status/ alcohol status, diabetes and dyslipidemia diseases.

# Table S 10. Stratified analysis of the association between essential minerals and acute pancreatitis risk by smoking status.

| **Variable** | **Smoking status** | | | | | | **Alcohol status** | | | | | |
| --- | --- | --- | --- | --- | --- | --- | --- | --- | --- | --- | --- | --- |
|  | **Never** | | **Previous** | | **Current** | | **Never** | | **Previous** | | **Current** | |
|  | HR (95%CI) | P | HR (95%CI) | P | HR (95%CI) | P | HR (95%CI) | P | HR (95%CI) | P | HR (95%CI) | P |
| Calcium | 0.97 (0.88, 1.06) | 0.503 | 0.91 (0.82, 1.01) | 0.087 | 0.98 (0.81, 1.19) | 0.863 | 0.89 (0.65, 1.21) | 0.459 | 1.17 (0.92, 1.49) | 0.193 | 0.94 (0.87, 1.00) | 0.068 |
| Chloride | 1.01 (0.91, 1.11) | 0.915 | 1.01 (0.92, 1.12) | 0.776 | 0.99 (0.82, 1.18) | 0.900 | 1.23 (0.98, 1.54) | 0.070 | 1.32 (1.04, 1.66) | 0.021 | 0.98 (0.92, 1.05) | 0.583 |
| Copper | 0.92 (0.83, 1.02) | 0.123 | 0.87 (0.78, 0.97) | 0.016 | 1.08 (0.91, 1.27) | 0.389 | 1.01 (0.77, 1.33) | 0.919 | 1.05 (0.82, 1.35) | 0.695 | 0.91 (0.84, 0.97) | 0.008 |
| Iodine | 1.05 (0.96, 1.14) | 0.310 | 0.91 (0.81, 1.01) | 0.069 | 1.17 (1.01, 1.36) | 0.034 | 1.05 (0.79, 1.40) | 0.722 | 1.20 (1.00, 1.44) | 0.045 | 1.00 (0.93, 1.06) | 0.890 |
| Iron | 0.94 (0.85, 1.03) | 0.193 | 0.91 (0.82, 1.00) | 0.058 | 1.07 (0.90, 1.29) | 0.440 | 1.01 (0.76, 1.33) | 0.945 | 1.11 (0.89, 1.39) | 0.346 | 0.92 (0.86, 0.99) | 0.027 |
| Magnesium | 0.92 (0.84, 1.02) | 0.107 | 0.89 (0.80, 0.99) | 0.029 | 1.04 (0.86, 1.26) | 0.666 | 0.80 (0.58, 1.12) | 0.201 | 1.09 (0.88, 1.36) | 0.438 | 0.92 (0.85, 0.98) | 0.014 |
| Manganese | 0.92 (0.83, 1.01) | 0.081 | 0.88 (0.80, 0.98) | 0.021 | 1.04 (0.86, 1.27) | 0.670 | 0.77 (0.54, 1.08) | 0.126 | 1.11 (0.86, 1.44) | 0.412 | 0.91 (0.85, 0.98) | 0.009 |
| Phosphorus | 0.99 (0.91, 1.09) | 0.880 | 0.92 (0.83, 1.01) | 0.094 | 1.05 (0.88, 1.26) | 0.587 | 0.99 (0.76, 1.31) | 0.968 | 1.14 (0.92, 1.40) | 0.225 | 0.95 (0.89, 1.02) | 0.183 |
| Potassium | 0.97 (0.88, 1.06) | 0.482 | 0.97 (0.87, 1.07) | 0.492 | 1.07 (0.89, 1.30) | 0.453 | 0.87 (0.64, 1.18) | 0.366 | 1.11 (0.91, 1.34) | 0.302 | 0.97 (0.91, 1.04) | 0.396 |
| Selenium | 1.05 (0.96, 1.14) | 0.291 | 1.00 (0.91, 1.11) | 0.929 | 1.14 (0.96, 1.34) | 0.129 | 1.12 (0.89, 1.41) | 0.347 | 1.20 (0.95, 1.51) | 0.127 | 1.03 (0.96, 1.09) | 0.432 |
| Sodium | 1.01 (0.92, 1.11) | 0.804 | 1.02 (0.92, 1.12) | 0.757 | 0.98 (0.82, 1.18) | 0.856 | 1.26 (1.01, 1.58) | 0.045 | 1.32 (1.03, 1.68) | 0.028 | 0.98 (0.92, 1.05) | 0.639 |
| Zinc | 0.98 (0.90, 1.08) | 0.737 | 0.92 (0.84, 1.02) | 0.132 | 0.99 (0.82, 1.19) | 0.899 | 1.02 (0.76, 1.37) | 0.909 | 1.10 (0.86, 1.40) | 0.447 | 0.95 (0.88, 1.01) | 0.107 |

HR: hazard ratio; CI: confidence interval. Model 2 were further adjusted with age, sex, ethnicity, body mass index (BMI), Townsend deprivation index (TDI), annual household income before tax, Metabolic Equivalent of Task (MET), smoking status/ alcohol status, diabetes and dyslipidemia diseases.

# Table S11. Stratified analysis of the association between essential minerals and acute risk of chronic pancreatitis and others by smoking status.

| **Variable** | **Smoking status** | | | | | | **Alcohol status** | | | | | |
| --- | --- | --- | --- | --- | --- | --- | --- | --- | --- | --- | --- | --- |
|  | **Never** | | **Previous** | | **Current** | | **Never** | | **Previous** | | **Current** | |
|  | HR (95%CI) | P | HR (95%CI) | P | HR (95%CI) | P | HR (95%CI) | P | HR (95%CI) | P | HR (95%CI) | P |
| Calcium | 1.01 (0.91, 1.10) | 0.915 | 0.97 (0.87, 1.07) | 0.508 | 1.05 (0.89, 1.24) | 0.556 | 1.02 (0.78, 1.33) | 0.879 | 1.00 (0.75, 1.34) | 0.984 | 0.99 (0.93, 1.06) | 0.881 |
| Chloride | 0.97 (0.88, 1.08) | 0.616 | 1.01 (0.92, 1.11) | 0.841 | 0.97 (0.82, 1.15) | 0.722 | 1.13 (0.86, 1.47) | 0.380 | 1.26 (0.96, 1.66) | 0.098 | 0.97 (0.91, 1.04) | 0.401 |
| Copper | 1.05 (0.96, 1.14) | 0.302 | 1.03 (0.94, 1.12) | 0.535 | 1.07 (0.92, 1.24) | 0.406 | 1.02 (0.78, 1.32) | 0.902 | 1.14 (0.90, 1.45) | 0.280 | 1.03 (0.97, 1.10) | 0.294 |
| Iodine | 1.04 (0.95, 1.14) | 0.368 | 0.96 (0.87, 1.06) | 0.419 | 1.07 (0.91, 1.26) | 0.391 | 1.22 (0.98, 1.51) | 0.075 | 1.01 (0.74, 1.37) | 0.962 | 1.00 (0.93, 1.06) | 0.931 |
| Iron | 1.00 (0.91, 1.10) | 0.969 | 1.05 (0.95, 1.15) | 0.330 | 1.02 (0.86, 1.21) | 0.854 | 0.99 (0.76, 1.31) | 0.966 | 1.17 (0.91, 1.51) | 0.225 | 1.02 (0.95, 1.09) | 0.654 |
| Magnesium | 0.98 (0.89, 1.08) | 0.748 | 1.02 (0.93, 1.13) | 0.652 | 1.05 (0.89, 1.25) | 0.552 | 0.98 (0.75, 1.28) | 0.873 | 1.13 (0.87, 1.45) | 0.359 | 1.00 (0.94, 1.07) | 0.958 |
| Manganese | 0.99 (0.90, 1.09) | 0.809 | 0.99 (0.90, 1.10) | 0.889 | 1.04 (0.87, 1.24) | 0.673 | 0.88 (0.65, 1.18) | 0.389 | 1.01 (0.76, 1.34) | 0.947 | 1.00 (0.94, 1.07) | 0.932 |
| Phosphorus | 1.03 (0.93, 1.13) | 0.583 | 1.00 (0.91, 1.10) | 0.988 | 1.03 (0.87, 1.22) | 0.739 | 1.04 (0.81, 1.34) | 0.742 | 1.14 (0.88, 1.49) | 0.313 | 1.00 (0.94, 1.07) | 0.924 |
| Potassium | 1.01 (0.92, 1.10) | 0.890 | 1.08 (0.98, 1.18) | 0.113 | 1.06 (0.90, 1.26) | 0.478 | 1.03 (0.81, 1.32) | 0.797 | 1.13 (0.88, 1.46) | 0.328 | 1.04 (0.97, 1.11) | 0.289 |
| Selenium | 1.04 (0.95, 1.14) | 0.419 | 0.96 (0.87, 1.06) | 0.422 | 1.11 (0.95, 1.30) | 0.179 | 1.15 (0.93, 1.43) | 0.194 | 0.95 (0.68, 1.33) | 0.767 | 1.00 (0.94, 1.07) | 0.886 |
| Sodium | 0.99 (0.90, 1.09) | 0.857 | 1.01 (0.91, 1.11) | 0.883 | 0.94 (0.79, 1.12) | 0.519 | 1.16 (0.89, 1.51) | 0.261 | 1.26 (0.96, 1.66) | 0.095 | 0.97 (0.91, 1.04) | 0.403 |
| Zinc | 1.00 (0.90, 1.10) | 0.936 | 1.07 (0.97, 1.17) | 0.182 | 0.97 (0.82, 1.16) | 0.759 | 0.96 (0.72, 1.28) | 0.807 | 1.19 (0.93, 1.54) | 0.173 | 1.01 (0.95, 1.08) | 0.717 |

HR: hazard ratio; CI: confidence interval. Model 2 were further adjusted with age, sex, ethnicity, body mass index (BMI), Townsend deprivation index (TDI), annual household income before tax, Metabolic Equivalent of Task (MET), smoking status/ alcohol status, diabetes and dyslipidemia diseases.

# Table S12. Stratified analysis of the association between essential minerals and pancreatic disease risk by diabetes disease.

| **Variable** | **Pancreatic Cancer** | | | | **Acute Pancreatitis** | | | | **Chronic Pancreatitis and others** | | | |
| --- | --- | --- | --- | --- | --- | --- | --- | --- | --- | --- | --- | --- |
|  | **No** | | **Yes** | | **No** | | **Yes** | | **No** | | **Yes** | |
|  | HR (95%CI) | P | HR (95%CI) | P | HR (95%CI) | P | HR (95%CI) | P | HR (95%CI) | P | HR (95%CI) | P |
| Calcium | 1.01 (0.88, 1.15) | 0.884 | 0.84 (0.55, 1.29) | 0.437 | 0.96 (0.90, 1.03) | 0.216 | 0.80 (0.61, 1.05) | 0.112 | 0.98 (0.91, 1.05) | 0.500 | 1.19 (0.99, 1.42) | 0.069 |
| Chloride | 0.97 (0.84, 1.11) | 0.628 | 1.08 (0.75, 1.56) | 0.693 | 1.00 (0.94, 1.07) | 0.968 | 1.12 (0.91, 1.39) | 0.288 | 0.96 (0.90, 1.03) | 0.254 | 1.23 (1.04, 1.45) | 0.013 |
| Copper | 1.02 (0.90, 1.16) | 0.747 | 1.11 (0.82, 1.50) | 0.501 | 0.93 (0.87, 1.00) | 0.042 | 0.82 (0.62, 1.08) | 0.160 | 1.03 (0.96, 1.09) | 0.425 | 1.13 (0.99, 1.28) | 0.070 |
| Iodine | 1.13 (1.02, 1.26) | 0.015 | 1.14 (0.84, 1.55) | 0.394 | 1.02 (0.96, 1.09) | 0.582 | 0.89 (0.68, 1.17) | 0.410 | 0.99 (0.93, 1.06) | 0.816 | 1.13 (0.97, 1.32) | 0.120 |
| Iron | 1.04 (0.91, 1.19) | 0.529 | 0.88 (0.58, 1.34) | 0.554 | 0.94 (0.87, 1.00) | 0.059 | 0.98 (0.77, 1.25) | 0.882 | 1.01 (0.94, 1.08) | 0.775 | 1.12 (0.95, 1.32) | 0.167 |
| Magnesium | 0.99 (0.86, 1.13) | 0.838 | 0.91 (0.60, 1.38) | 0.658 | 0.92 (0.86, 0.99) | 0.022 | 0.92 (0.71, 1.18) | 0.494 | 1.00 (0.93, 1.07) | 0.934 | 1.10 (0.93, 1.31) | 0.263 |
| Manganese | 0.91 (0.79, 1.05) | 0.193 | 0.93 (0.61, 1.42) | 0.733 | 0.92 (0.86, 0.99) | 0.018 | 0.85 (0.65, 1.12) | 0.245 | 0.99 (0.92, 1.05) | 0.673 | 1.11 (0.92, 1.32) | 0.279 |
| Phosphorus | 1.03 (0.90, 1.18) | 0.652 | 0.81 (0.54, 1.23) | 0.323 | 0.97 (0.91, 1.04) | 0.376 | 0.94 (0.74, 1.19) | 0.611 | 0.99 (0.93, 1.06) | 0.821 | 1.17 (1.00, 1.38) | 0.052 |
| Potassium | 0.99 (0.86, 1.13) | 0.858 | 0.92 (0.62, 1.37) | 0.689 | 0.97 (0.91, 1.04) | 0.427 | 1.01 (0.80, 1.27) | 0.927 | 1.03 (0.97, 1.10) | 0.299 | 1.09 (0.92, 1.29) | 0.305 |
| Selenium | 1.13 (1.01, 1.27) | 0.031 | 1.12 (0.80, 1.57) | 0.525 | 1.04 (0.98, 1.11) | 0.210 | 1.05 (0.83, 1.32) | 0.709 | 0.99 (0.93, 1.06) | 0.802 | 1.15 (1.00, 1.33) | 0.055 |
| Sodium | 0.97 (0.84, 1.11) | 0.628 | 1.12 (0.78, 1.61) | 0.553 | 1.00 (0.94, 1.07) | 0.910 | 1.13 (0.92, 1.40) | 0.246 | 0.96 (0.90, 1.03) | 0.269 | 1.24 (1.05, 1.46) | 0.011 |
| Zinc | 1.08 (0.95, 1.23) | 0.256 | 0.83 (0.55, 1.25) | 0.373 | 0.95 (0.89, 1.02) | 0.153 | 1.04 (0.83, 1.29) | 0.740 | 1.02 (0.95, 1.09) | 0.648 | 1.06 (0.88, 1.26) | 0.561 |

HR: hazard ratio; CI: confidence interval. Model 2 were further adjusted with age, sex, ethnicity, body mass index (BMI), Townsend deprivation index (TDI), annual household income before tax, Metabolic Equivalent of Task (MET), smoking status, alcohol status, dyslipidemia diseases.

# Table S13. Stratified analysis of the association between essential minerals and pancreatic disease risk by dyslipidemia disease.

| **Variable** | **Pancreatic Cancer** | | | | **Acute Pancreatitis** | | | | **Chronic Pancreatitis and others** | | | |
| --- | --- | --- | --- | --- | --- | --- | --- | --- | --- | --- | --- | --- |
|  | **No** | | **Yes** | | **No** | | **Yes** | | **No** | | **Yes** | |
|  | HR (95%CI) | P | HR (95%CI) | P | HR (95%CI) | P | HR (95%CI) | P | HR (95%CI) | P | HR (95%CI) | P |
| Calcium | 1.10 (0.94, 1.29) | 0.245 | 0.84 (0.68, 1.03) | 0.100 | 0.96 (0.88, 1.04) | 0.314 | 0.94 (0.85, 1.04) | 0.220 | 1.02 (0.94, 1.11) | 0.575 | 0.96 (0.86, 1.06) | 0.422 |
| Chloride | 0.94 (0.79, 1.13) | 0.517 | 1.01 (0.84, 1.22) | 0.907 | 1.05 (0.96, 1.13) | 0.278 | 0.96 (0.87, 1.06) | 0.449 | 1.04 (0.96, 1.12) | 0.360 | 0.92 (0.83, 1.02) | 0.120 |
| Copper | 1.08 (0.94, 1.25) | 0.263 | 0.94 (0.76, 1.15) | 0.529 | 0.90 (0.82, 0.98) | 0.019 | 0.96 (0.86, 1.06) | 0.386 | 1.07 (1.00, 1.15) | 0.057 | 0.99 (0.90, 1.09) | 0.858 |
| Iodine | 1.20 (1.11, 1.31) | 0.000 | 0.93 (0.76, 1.14) | 0.480 | 1.04 (0.96, 1.12) | 0.327 | 0.97 (0.87, 1.07) | 0.515 | 1.03 (0.96, 1.11) | 0.429 | 0.97 (0.88, 1.08) | 0.588 |
| Iron | 1.08 (0.92, 1.26) | 0.360 | 0.94 (0.77, 1.15) | 0.564 | 0.93 (0.85, 1.01) | 0.099 | 0.95 (0.86, 1.05) | 0.351 | 1.06 (0.98, 1.14) | 0.173 | 0.97 (0.88, 1.08) | 0.588 |
| Magnesium | 1.06 (0.90, 1.24) | 0.471 | 0.85 (0.69, 1.05) | 0.130 | 0.92 (0.84, 1.00) | 0.046 | 0.93 (0.84, 1.03) | 0.180 | 1.03 (0.95, 1.12) | 0.439 | 0.97 (0.88, 1.08) | 0.602 |
| Manganese | 1.03 (0.88, 1.21) | 0.708 | 0.74 (0.59, 0.92) | 0.007 | 0.89 (0.82, 0.97) | 0.011 | 0.95 (0.85, 1.05) | 0.317 | 1.00 (0.92, 1.09) | 0.982 | 0.99 (0.89, 1.10) | 0.904 |
| Phosphorus | 1.11 (0.95, 1.29) | 0.194 | 0.85 (0.69, 1.05) | 0.133 | 1.00 (0.92, 1.08) | 0.968 | 0.93 (0.84, 1.03) | 0.149 | 1.05 (0.97, 1.14) | 0.199 | 0.95 (0.86, 1.06) | 0.353 |
| Potassium | 0.98 (0.83, 1.16) | 0.852 | 0.96 (0.79, 1.17) | 0.707 | 0.97 (0.89, 1.05) | 0.491 | 0.99 (0.89, 1.09) | 0.770 | 1.08 (1.01, 1.17) | 0.029 | 0.97 (0.87, 1.07) | 0.545 |
| Selenium | 1.23 (1.10, 1.37) | 0.000 | 0.93 (0.75, 1.14) | 0.466 | 1.08 (1.01, 1.16) | 0.028 | 0.97 (0.87, 1.07) | 0.550 | 1.05 (0.97, 1.13) | 0.238 | 0.95 (0.85, 1.06) | 0.372 |
| Sodium | 0.96 (0.80, 1.14) | 0.629 | 1.00 (0.83, 1.21) | 0.974 | 1.06 (0.98, 1.14) | 0.168 | 0.95 (0.86, 1.05) | 0.329 | 1.04 (0.96, 1.13) | 0.335 | 0.92 (0.83, 1.02) | 0.126 |
| Zinc | 1.11 (0.95, 1.30) | 0.184 | 0.95 (0.78, 1.16) | 0.623 | 0.95 (0.87, 1.03) | 0.210 | 0.98 (0.89, 1.08) | 0.653 | 1.06 (0.98, 1.14) | 0.145 | 0.96 (0.87, 1.07) | 0.448 |

HR: hazard ratio; CI: confidence interval. Model 2 were further adjusted with age, sex, ethnicity, body mass index (BMI), Townsend deprivation index (TDI), annual household income before tax, Metabolic Equivalent of Task (MET), smoking status, alcohol status, diabetes diseases.
